# Evaluating the performance of EEG based on ANN to predict the effectiveness of tDCS combined evaluative conditioning on obsession symptoms reduction in contamination OCD patients: Secondary analysis of data from a randomized controlled trial

**DOI:** 10.1371/journal.pone.0354614

**Published:** 2026-08-20

**Authors:** Fateme Asadollahzadeh Shamkhal, Ali Moghimi, Hamid Reza Kobravi, Javad Salehi Fadardi, Faezeh Raeis Al Mohaddesin

**Affiliations:** 1 Rayan Research Center for Neuroscience & Behavior, Department of Biology, Faculty of Science, Ferdowsi University of Mashhad, Mashhad, Iran; 2 Departmen of Biomedical Engineering, Faculty of Electrical Engineering, Islamic Azad University of Mashhad, Mashhad, Iran; 3 School of Community and Global Health, Claremont Graduate University, Claremont, United States of America; 4 Department of Psychology, Faculty of Education and Psychology, Ferdowsi University of Mashhad, Mashhad, Iran; University of Minho: Universidade do Minho, PORTUGAL

## Abstract

Over 40% of Obsessive-Compulsive Disorder (OCD) patients do not respond to common treatments. This study was a secondary analysis of data from a randomized controlled trial, for predictability of effectiveness of Transcranial Direct Current Stimulation (tDCS) with Contamination-Based OCD (C-OCD) using artificial neural networks (ANN) and electroencephalography (EEG) signals. Out of 54 C-OCD patients, 48 were randomized into 4 groups. Using a 2 × 2 factorial design, each group received a combined intervention (real or sham) based on tDCS and Disgust Reduction Evaluative Conditioning (DREC) in 10 sessions. Evaluations included the Yale-Brown Obsessive-Compulsive Scale (Y-BOCS) and EEG recordings at rest (eyes open). Among the various features extracted from EEG (Fuzzy Synchronization Likelihood (FSL), Power Spectrum, and Recurrence Quantification Analysis (RQA), the Relief algorithm identified appropriate features based on intervention effectiveness for each frequency band and group allocation. The obsessive symptoms reduction was predicted using appropriate features, the fully connected feedforward network and Radial Basis Functions (RBF) networks. The ANN inputs were the appropriate features extracted from the EEG signal before the interventions. To predict the effectiveness level, the Y-BOCS change score (pre- to post-intervention) was given to the models as the desired output. Both networks had the best results for the first three proposed Relief features. The fully connected feedforward network optimized by Gray Wolf Optimizer (GWO) achieved the best predictive performance with an average RMSE 0.57 ± 0.4. Based on the calculated error and comparison with Y-BOCS change intervals, EEG data from C-OCD patients combined with ANN effectively predicted the extent to which each type of intervention can change patients’ Y-BOCS score. So, the therapist can decide whether to perform or select each type of intervention for the patient before starting treatment. Our findings confirm the feasibility of using pre-intervention EEG signals combined with ANN to predict individual responses in C-OCD patients.

## Introduction

Obsessive-Compulsive Disorder (OCD) is defined as obsessive thoughts or compulsive behaviors. Obsessive thoughts are characterized by increased anxiety and distress, and compulsive behaviors are performed in response to obsessions [1,2]. OCD can be very severe and debilitating, and can manifest itself in a variety of ways. OCD disorder is common, and about 2.5 to 3% of the entire population has this disorder. OCD is a complex neurological disorder that involves a complex network of neurotransmitters, including serotonin, dopamine, glutamate, and GABA [3].

Over 25 years of non-invasive research using functional brain imaging techniques has demonstrated that abnormal patterns exist in a specific area of the brain called the Cortico-Striato-Thalamo-Cortical (CSTC) circuit. These studies have paved the way for exploring the neurobiology of OCD. The CSTC circuit, also known as the Frontostriatal or Corticostriatal model, consists of two pathways: direct and indirect. Disturbances in the CSTC circuits have been observed in the Orbitofrontal Cortex (OFC), Dorsolateral Prefrontal Cortex (DLPFC), and caudate nucleus. This circuit is thought to be related to executive cognitive functions [4]. One of the most common types of obsessive-compulsive disorder is Contamination-Based OCD (C-OCD). At least 50% of all OCD sufferers have symptoms of contamination-type obsessive-compulsive disorder [1,5]. People with C-OCD experience anxiety when exposed to pollutants. There is also evidence that disgust plays a role in this anxiety. Also, C-OCD may reflect a disorder in the appraisal and processing of disgust [5–7]. It should be noted that following the COVID-19 pandemic, an increase in the severity of contamination obsessions and compulsions in OCD patients and even the prevalence of contamination obsessions among healthy individuals has been reported in some studies due to frequent hand washing and hygiene practices to prevent COVID infection [8].

The first-line treatment for OCD is pharmacotherapy using non-selective serotonin reuptake inhibitors (SSRIs) and cognitive behavioral therapy (CBT), either alone or in combination [9]. In addition, invasive brain stimulation methods such as deep brain stimulation (DBS) or non-invasive methods such as Transcranial magnetic stimulation (TMS), Transcranial direct current stimulation (tDCS), and Electroconvulsive therapy (ECT) are also among the proposed treatment methods for this disease. These treatments can be applied individually with specific intensity, stimulation time, and frequency, targeting specific brain areas [10]. It should be noted that between 40–60% of patients do not respond adequately to these first-line treatments (pharmacotherapy and psychology), and about 80% of people have relapses after taking medications [11]. So, in OCD patients who have an elevated level of resistance to treatment [12], predicting the level of effectiveness before starting treatment in OCD patients is important.

The main purpose of this study was to predict the effectiveness of the intervention using electroencephalography (EEG) signals before performing a therapeutic intervention in C-OCD patients. Prediction can help therapists choose the best and most appropriate treatment method specifically for each patient. On the other hand, choosing the best treatment strategy for each individual can prevent the time and material cost spent on ineffective treatment interventions. Also, the patient expectation of the result of the treatment strategy comes closer to reality.

First, the study assessed the effectiveness of tDCS and Disgust Reduction Evaluative Conditioning (DREC), a new type of intervention, in C-OCD patients [13]. Second, the linear and nonlinear features extracted from the EEG signal were examined, and the best features were selected using the Relief algorithm. So that the selected features can reveal the effect of tDCS and DREC intervention in OCD patients, independently of the Yale-Brown Obsessive-Compulsive Scale (Y-BOCS) score or other common questionnaires. Finally, the level of effectiveness of the interventions was predicted using the appropriate features extracted from the EEG signal and combining the fully connected feedforward network with the Gray Wolf Optimizer (GWO).

Within the general framework of the study described above, we proposed the hypothesis that integrating EEG processing with machine learning methods (black box models) will enable accurate prediction of intended effectiveness.

## Materials and methods

### Subjects

This study was a secondary analysis of data from a randomized controlled trial that examined the predictability of the effectiveness of tDCS with C-OCD using EEG signals and Artificial Neural Network (ANN). The trial protocol has been published in a previous study [13], and is briefly described below.

The 48 participating patients were between 18–55 years of age (31.1 ± 9.57) and included 7 males and 41 females. Patients participated in this study based on referrals from a psychiatrist or through project information, and voluntarily after interviewing and examining. The selection criteria for patients in the first step were clinical diagnosis based on the Diagnostic and Statistical Manual of Mental Disorders, Fifth Edition, Text Revision (DSM-5-TR) and being in the C-OCD subgroup. Other criteria for entry into the study included age between 18–55 years, ability to read, write, and work with a computer. Exclusion criteria included addiction and substance abuse, the presence of psychotic symptoms or other psychiatric disorders with high symptom severity, specific physical conditions that made it impossible for the patient to participate in sessions and assessments, invalid response on baseline tests and a positive response to at least one item of the Transcranial Electrical Stimulation (tES) Screening Questionnaire [14].

### Study design & interventions

Forty-eight C-OCD patients were randomly assigned to one of four arms (1:1:1:1) using a blocked randomization list in blocks of four that was generated by sealedenvelope.com. They assigned to four parallel groups (n = 12) to receive ten sessions of active or sham DREC and tDCS in a 2x2 factorial design: (1) active DREC + active tDCS, (2) active DREC + sham tDCS, (3) sham DREC + active tDCS, and (4) sham DREC + sham tDCS. The interventions were implemented in 10 sessions (5 days per week). The tDCS and DREC interventions were administered simultaneously.

Sham interventions were considered to separate the exposure effect from the DREC effect and monitor the placebo effect. After the completion of 10 interventional sessions, and also after a 2-month follow-up, assessments were done. All groups had equal intervention amounts (activity, number of sessions, and equal time) and similar assessments.

For applying tDCS, the cathode electrode was placed on the left OFC (or FP1) and the anode on the right cerebellum (3 cm below the Inion and 1 cm from the midline to the right) and was performed in ten 20-minute sessions with a current of 2 mA using the ActivaDose II device. In the sham group, the electrodes were placed on the same areas. However, the applied current was 0.1 mA. The dimensions of the cathode electrode used were 7 × 5, and the anode was 10 × 10 cm square. This montage is similar to that of Bation et al. [15], but due to having a more focal cathodal effect over the left OFC, the size of the anode electrode (reference) has been increased.

Inspired by the general principles of the intervention used in the Kosinski study [16] and after making changes appropriate to the research objectives, the DREC program was designed as a computer program in the PsychoPy Software. The program was in two active (real) and control (sham) forms in four levels of difficulty. In the active DREC, 30 pollution-related images were used as Conditioned Stimulus (CS) and 30 pleasant images as Unconditioned Stimulus (US) in this program, each CS was always accompanied by a specific US; and 30 CS-US pairs were obtained from their pairing.

The sham version of this program was designed the same as the active version, except that 30 neutral-valence pictures were substituted as US. In total, each session lasted about 15 minutes and included 240 trials.

Pre‑, post‑, and follow‑up assessments were conducted through EEG signal and cognitive tests and self‑report measures. The EEG signal was recorded in the resting state. Recording was performed as a 19-channel EEG with a Mitsar model 202 device based on the 10–20 standard in the resting state with eyes-open (EO) and eyes-closed (EC) before, after the interventions, and at 2 months follow-up, at least for 3 minutes. Sampling frequency was 500 Hz. Cognitive tests included the Dot-probe test (DPT) and the Emotional Go/NoGo task (EGNT), and Self‑report measures were included Y-BOCS, Padua Inventory-Washington State University Revision (PI-WSUR), Disgust Scale-Revised (DS-R), Beck Depression Inventory-II (BDI-II), and Beck Anxiety Inventory (BAI).

**Fig 1:**
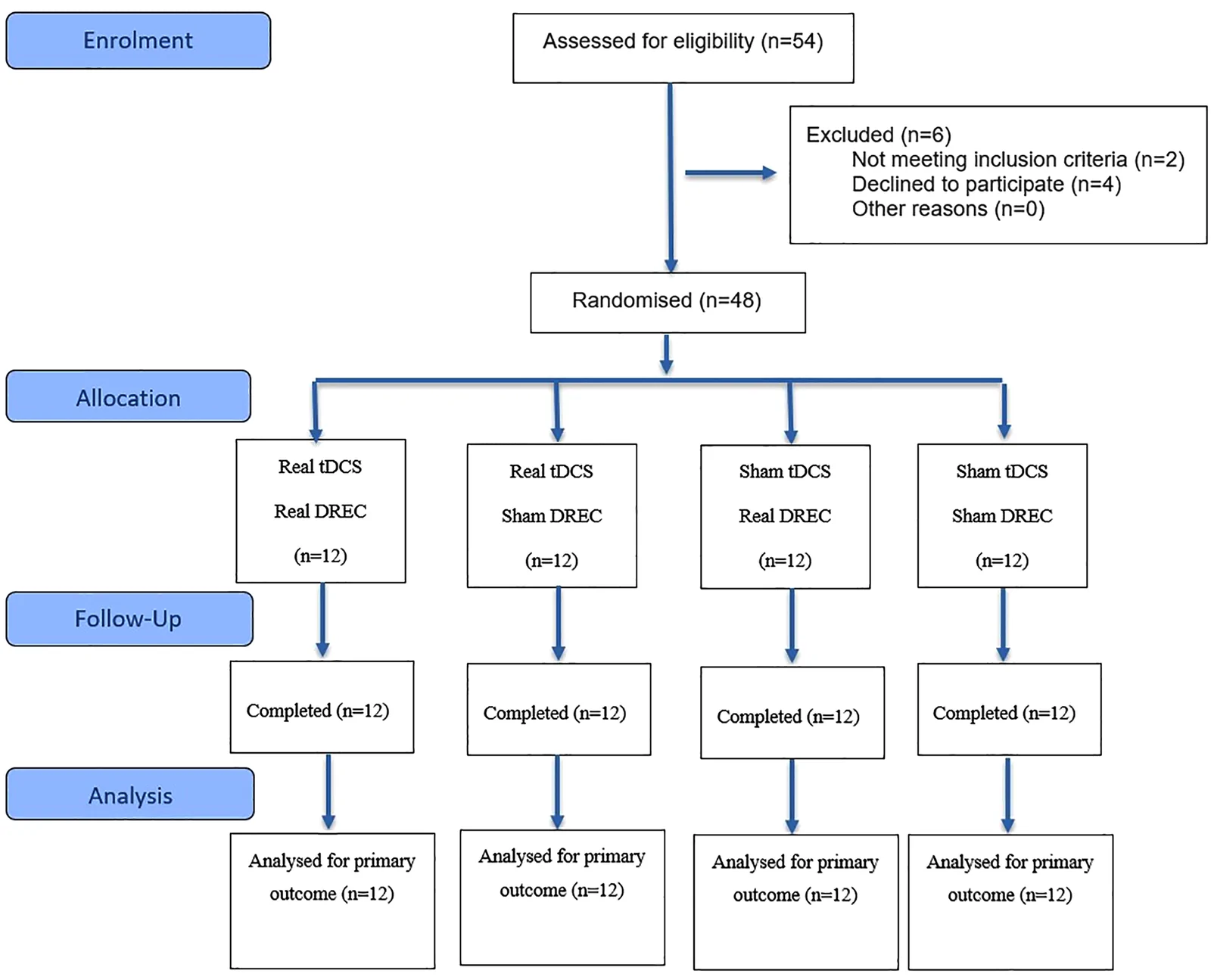
CONSORT flowchart.

Notably, the tDCS has been reported to be safe for OCD symptom reduction in previous RCTs (Randomized Controlled Trials) [15,17]. However, it was predicted that if a participant reports any problem, the tDCS will be stopped and the trial will be discontinued for that participant. Also, if more than two participants report serious side effects, the tDCS will be discontinued. To review the implementation of the trial, the Trial Management Group (TMG) and the Trial Steering Committee (TSC) met every week and every month, respectively. The TMG was responsible for ensuring that the trial was conducted efficiently and effectively, and the TSC provided reports to the ethics committee and funders. The principal investigator was responsible for enrolling participants and assigning them to interventions. Fig 1 shows the design of the trial.

The study protocol was a single-blind design. Participants were blind to whether one or both interventions were sham or active until the end of follow-up assessments. It should be pointed out that the sample size was calculated using the G*Power 3.1 software. In this study, 0.4, 0.8, 0.05, 4, and 3 were set as the effect size, the statistical power, the alpha value, the number of groups, and the number of measurements, respectively. Additionally, the F tests and the MANOVA, as a subtype of F tests family, were utilized. Accordingly, the sample size of 46 participants was calculated [13].

All experimental protocols were approved by the Biomedical Ethics Committee of Ferdowsi University of Mashhad for the psychological part of experiments (ID Number: IR.UM.REC.1400.350). According to the Biomedical Ethics Committee instructions, a written and signed consent form was completed by all participants (all forms saved in our research documents). The trial was registered on the ClinicalTrials.gov website (identifier: NCT05907369). No important changes to outcomes or analyses have been made since the protocol submission to ClinicalTrials.gov. Also, the recruitment period for human participants was from 20 January 2023–30 December 2023. The trial was conducted in the Cognitive Science Laboratory of Ferdowsi University of Mashhad. For more details about interventions and assessments, see previous research [13].

In this study, only Y-BOCS scores and EO EEG signals before and after interventions were analyzed. It should be noted that this study has been approved by the Biomedical Ethics Committee of Ferdowsi University of Mashhad for the electrophysiological part of experiments (ID Number: IR.UM.REC.1401.006).

### EEG data & processing

For EEG preprocessing and removing artifacts and noises (such as motion artifacts, blinking, etc.), a high-pass (0.5 Hz) and low-pass (45 Hz) IIR (Infinite Impulse Response) filter of degree 40 was used, as well as using Independent Component Analysis (ICA) in MATLAB (https://www.mathworks.com/products/matlab.html). A notch filter was also used to remove power line noise. The data were also filtered and separated for the entire frequency spectrum, including delta (0.5–4 Hz), theta (4–8 Hz), alpha (8–12 Hz), beta (12–30 Hz), and gamma (30–45 Hz). Anonymized EEG data are provided in S1 and S2 Datasets.

### Feature extraction

In this study, features of the EEG signal have been considered to describe the dynamics of brain signals in the time, frequency, and state space domains. They can also show the changes made after receiving interventions and the degree of response to treatment, and describe changes in clinical symptoms. Therefore, in this study, complexity analysis using the Recurrence Quantification Analysis (RQA) method, frequency domain analysis by examining power features (mean, median, peak, minimum, and maximum), and connectivity analysis by analyzing the Fuzzy Synchronization Likelihood (FSL) feature have been performed. All extracted features have been calculated separately for all frequency bands (delta, theta, alpha, beta and gamma).

To determine the power density of frequency components in a signal based on the Fourier transform, the periodogram method has been used. To calculate the power spectral density using the periodogram, the Fourier transform of the signal must first be obtained [18]. See S1 Appendix for Equations.

RQA analysis is a nonlinear method for investigating dynamics in dynamic systems and the complexity of variables, and includes a set of features for quantitative analysis of Recurrence Plots (RP). The Recurrence rate (RR) feature is a brain dynamics descriptor, where changes in it are interpreted as changes in the dynamics of brain interactions. Previous studies have shown that RQA is a suitable tool for studying brain dynamics induced by any brain stimulation [19].

The RQA features discussed in this research are

Recurrence rate (RR): The return rate is a measure of the density of returning points in the RP, which is defined as follows:


$$\begin{array}{c} RR=\frac{\text{1}}{N^{\text{2}}}\sum_{i,j=\text{0}}^{N}R_{i,j} \end{array}$$ (1)

In the above equation, the value of *R*_*i,j*_ is the mathematical expression of RP, which is expressed as follows:


$$\begin{array}{c} R_{i.j=\;}\Theta \left(\varepsilon_{i}-\left\|x_{i}-x_{j}\right\|\right),{\;x}_{i}\in {ℜ}^{m},i,j=\text{1}\ldots .N. \end{array}$$ (2)

where Θ is the Heaviside Step function, ||.|| is the norm, *N* is the total number of considered states, *x*_*i*_ is the considered state, and *ε*_*i*_ is the threshold distance.

The percentage of determinism (DET) is defined as the following equation:


$$\begin{array}{c} DET=\frac{\sum_{l=l_{min}}^{N}lP(l)}{\sum_{i,j}^{N}R_{i,j}} \end{array}$$ (3)

where *l* is the length of diagonal lines in the recurrence plot, *P(l*) is the number of diagonal lines with length equal to *l*, and *l*_*min*_ is the minimum length required to define a diagonal line.

The Length of longest diagonal line (*Lmax*) is defined by the following equation:


$$\begin{array}{c} L_{max}=\text{max}(\{l_{i};i=\text{1}\ldots N_{l}\}) \end{array}$$ (4)

RQA features were extracted with the CRP Toolbox implemented in MATLAB [20].

The dynamic connections and interactions between EEG channel signals are indicative of brain behavior and dynamics. Therefore, quantifying the dynamic interactions among recorded EEG channels can reveal various aspects of brain behavior change. The FSL can quantify these dynamics and connections between EEG signal channels.

The Synchronization Likelihood (SL) feature is one of the features that has found wide application in non-stationary systems and systems with high degree of nonlinearity in recent years. The SL feature allows measuring the interdependence in nonlinear systems. In this method, the similarity of patterns to each other is measured statistically, but the degree of similarity is not considered in decision-making, and it is determined logically whether the patterns are similar to a threshold or not. To solve this problem, a fuzzy part has also been added to this method, and for this reason, this solution is called FSL, which is more reliable than the SL method. In the FSL method, the state space of each time series is represented by embedded time-delay vectors, and to find similar dynamic states, reconstructed vectors that are similar in the state space must be searched. All Generalized Synchronization (GS) methods integrate time series synchronization into state space based on chaos theory and reconstruct the state space using Takens [21–23]. See S1 Appendix for more details about calculating FSL.

The final FSL relationship is obtained as the following relationship [23]:


$$\begin{array}{c} S_{\text{2},n}=\frac{\text{1}}{\;\text{2}\;P_{ref}(w\text{2}-w\text{1})}\sum_{\begin{array}{c} m=\text{1}\\ w\text{1}<|n-m|<w\text{2} \end{array}}^{N}\left[{\mu_{\text{1},n}(X_{\text{1},m})\mu }_{\text{2},n}(X_{\text{2},m})\right] \end{array}$$ (5)

### Feature selection

The appropriate features can make a meaningful distinction between brain dynamics before and after the intervention to show the dynamics caused by the interventions (tDCS and DREC), because the interventions, especially tDCS, are expected to directly affect the EEG characteristics. To implement this goal, a modified version of the well-known Relief algorithm, ReliefF, has been used, which has advantages over the Relief algorithm. This algorithm is suitable for noisy and incomplete data. It can also distinguish more than two data categories from each other. Feature selection in this algorithm is based on a statistical method and is based on the near hit and near miss distance criteria. To carry out this method, the pre-measurement data for all participants served as the first dataset, while the post-measurement data for all participants formed the second dataset. The neighbor search involved identifying the nearest “hit” and “miss” data points based on the established data labeling approach. Consequently, no data splitting or exclusion was performed. Essentially, the greater the difference between the pre- and post-measurement values of a particular feature, the higher its weight and rank determined by the Relief method. The higher weight indicates a high ability to separate classes by a particular feature. This approach facilitated the feature ranking process using the Relief method [24,25].

The algorithm was implemented in MATLAB software to distinguish the optimal features (i.e., those most influenced by the interventions) among the nine linear and nonlinear features extracted from the EEG signals in each group. The input to the ReliefF algorithm is a matrix that contains 9 features extracted from the EEG signal (before and after interventions), including power features (mean, median, peak, minimum, and maximum), FSL, and RQA features (RR, DET, Lmax). ReliefF was applied separately for each intervention group (4 groups) and each frequency spectrum (delta, theta, alpha, beta, and gamma). That is, the Relieff algorithm was run 5 × 4 times =20.

### Artificial neural networks

A black-box model refers to a machine learning model that operates as an opaque system where the internal workings of the model are not easily accessible or interpretable. These models make predictions based on input data, but the decision-making process and reasoning behind these predictions are not transparent to the user [26]. Given that the exact details of the effect of tDCS on brain function and molecular mechanisms are still largely unknown, a black-box model was used in this study [27,28].

**Fig 2:**
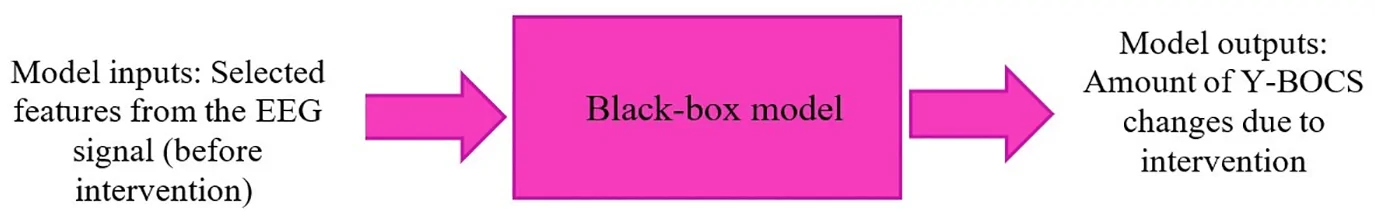
Proposed black-box model.

In this study, black-box models based on Radial Basis Function (RBF) and a model based on fully connected feedforward network were used to predict the level of effectiveness of interventions using EEG signals. Fig 2 shows the black-box model proposed in this study. The input to the model is the selected features of the EEG signal before the interventions, which were explained in the previous section. The expected output of the model is the amount of change in Y-BOCS scores. Changes were calculated based on the difference between the post-intervention Y-BOCS score and the pre-intervention Y-BOCS score. If the change rate is negative, it means a decrease in the patient’s obsessive symptoms, and if the change is positive, it indicates an increase in the patient’s obsessive symptoms after receiving the interventions.

RBF neural networks use radial basis functions as their activity functions. When an RBF network is used to perform a complex classification task, the problem is solved by nonlinearly transforming it into a high-dimensional space and then separating the classes in the output layer. The structure of this network consists of 3 layers. The first layer is the input. The second layer is the hidden layer, which takes a nonlinear transformation from the input space to the hidden (feature) space. The third layer is the output layer, which is a linear layer [29].

Equation 6 shows the structure of a shallow RBF network, which consists of a layer of RBF kernels followed by a fully connected linear layer. The linear layer combines the outputs of the RBF kernel based on the weight matrix W [30].


$$\begin{array}{c} \;y_{i}(x)=\sum_{i=1}^{k}w_{ij}.h_{i}\left(𝐱\right) \end{array}$$ (6)

The activation kernel *h*_*i*_*(x)* of neuron i is calculated by the square of the Mahalanobis distance between the center *C*_i_ and the input vector *x* through the activation function *f* as follows:


$$\begin{array}{c} h_{i}(𝐱)=f({\left\|x-c_{i}\right\|}_{R_{i}}^{2}) \end{array}$$ (7)

In a fully connected feedforward neural network, all neurons (nodes) within each layer are connected to every neuron in the adjacent forward layer, meaning that each node receives input from—and sends its output to—all nodes in the neighboring layers [29].

In this model, the number of layers was set to three.

The values of weights and bias in fully connected feedforward network are the most important parameters in training this network. To adjust these parameters and reduce the prediction error in fully connected feedforward network, meta-heuristic algorithms can be used. One of these algorithms is the Gray Wolf algorithm, which was introduced in 2014. In this study, this algorithm has been used to optimize the results [31,32].

## Results

### Y-BOCS assessment

All data from 48 participants were analyzed, and no serious side effects were reported in participants. A decrease in Y-BOCS score means an improvement in symptoms of obsessive-compulsive disorder (compulsion and obsession). Table 1 shows the demographics and the mean and standard deviation of Y-BOCS scores before and after the interventions for each group. Y-BOCS scores decreased in all groups after the intervention, but the decrease was not significant just in tDCS (sham)+ DREC (sham) (p = 0.7 (p > 0.05)). The normality of the data distribution was checked using the Shapiro–Wilk test. If the data distribution was normal, the Paired sample t test was used, and otherwise, the Wilcoxon signed-rank test was used. All statistical analyses were done using IBM SPSS Statistics for Windows version 26.0.

**Table 1:** Demographics and clinical characteristics for each group.

	Group 1: tDCS (sham) ± DREC (sham)	Group 2: tDCS ± DREC (sham)	Group 3: DREC ± tDCS (sham)	Group 4: DREC ± tDCS
**Age (Mean ± STD)**	26.67 ± 5.85	35.75 ± 10.50	24.50 ± 6.95	37.50 ± 7.76
**Gender (% Female)**	83.30	83.30	91.70	83.30
**Y-BOCS before intervention (Mean ± STD)**	24 ± 4.84	26.5 ± 3.8	23.75 ± 3.25	25.25 ± 4.07
**Y-BOCS after intervention (Mean ± STD)**	23.75 ± 6.34	21.91 ± 7.36*	16.92 ± 3.94 *	19.67 ± 3.06 *

* p < 0.05.

Also, Table 2 shows the Analysis of Variance (ANOVA) for the difference between post-intervention and pre-intervention Y-BOCS scores, which was considered the measure of change. Participants were divided into four groups based on the type of intervention received: Group 1 (sham DREC + sham tDCS), group 2 (sham DREC + active tDCS), group 3 (active DREC + sham tDCS), and group 4 (active DREC + active tDCS). The one-way ANOVA demonstrated a significant overall difference in Y-BOCS change scores among the four groups (F(3,44) = 7.016, p = 0.001). Post hoc analyses using the Games–Howell test indicated that group 1 showed significantly smaller reductions in Y-BOCS scores compared with groups 3 (p < 0.001) and 4 (p = 0.001), suggesting that the intervention in group 1 was less effective. The difference between group 1 and group 2 was not statistically significant (p = 0.099), and no significant differences were observed among groups 2, 3, and 4. Overall, these findings suggest that the intervention applied in Group 1 was less effective in alleviating obsessive–compulsive symptoms, whereas interventions in groups 3 and 4 produced the greatest improvements. Y-BOCS scores before and after intervention are provided in the S2 File.

**Table 2:** One-way ANOVA results for between-group differences in Y-BOCS change.

	Sum of Squares	df	Mean Square	F	Sig.
**Between Groups**	294.562	3	98.187	7.016	.001
**Within Groups**	615.750	44	13.994		
**Total**	910.313	47			

Improved individuals or treatment-responsive individuals were referred to as a reduction (≥1) Y-BOCS score decreased after receiving the mentioned interventions, and treatment-resistant individuals are those whose Y-BOCS score remained unchanged or increased (≥1) after the intervention. Therefore, the criteria for improvement and lack of improvement are defined in this study.

### Power assessment

In this study, the appropriate features were selected using the ReliefF algorithm. It was observed that the most suggested and repeated features by ReliefF were the median and peak power and FSL, respectively. Therefore, in order to further investigate the power feature, QEEG was plotted for all subjects across intervention groups and frequency bands (delta, theta, alpha, beta, and gamma). Power was calculated by using the periodogram method with the EEGLAB toolbox implemented in MATLAB [33].

**Fig 3:**
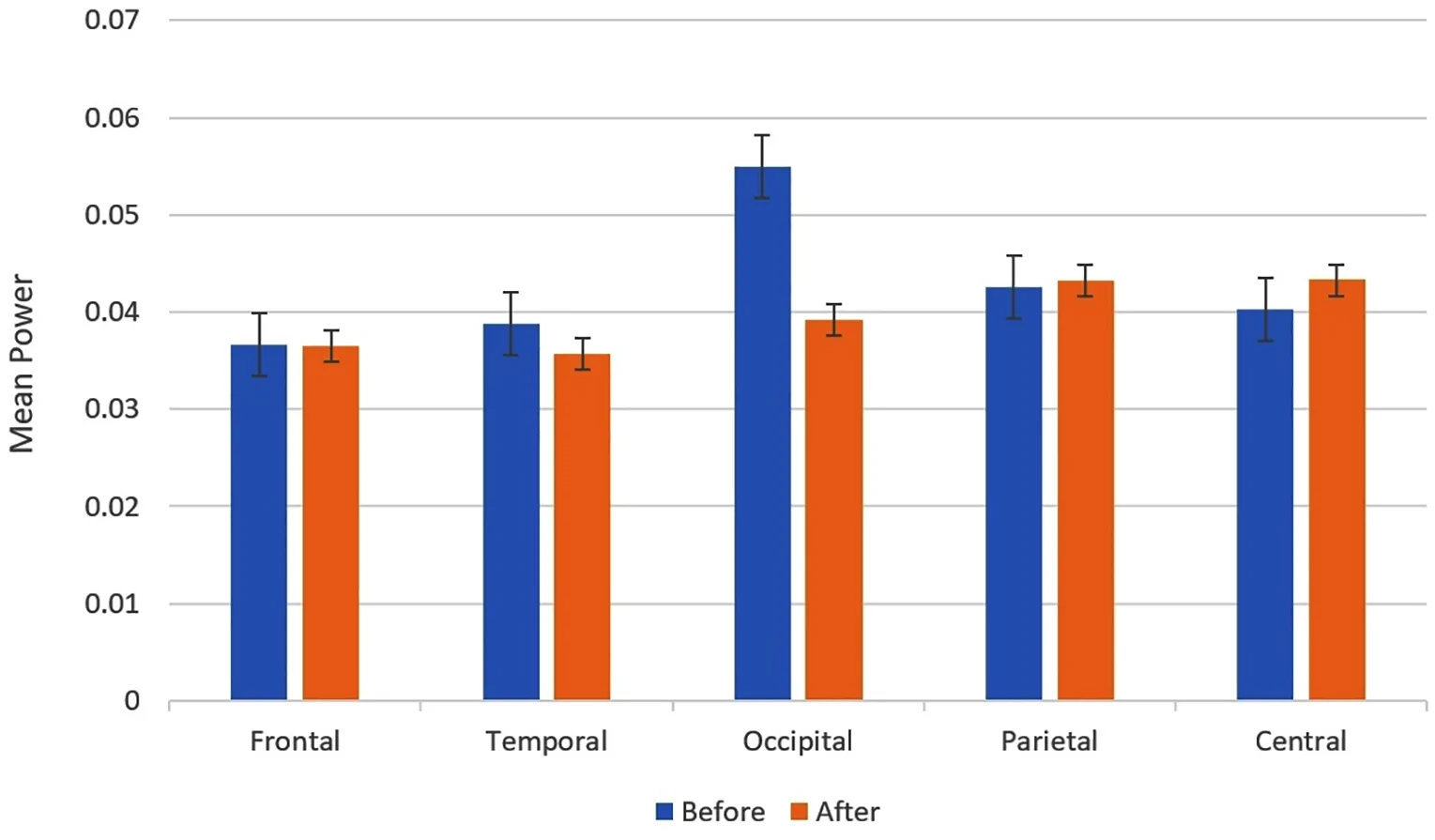
Comparison of mean power in brain lobes in treatment-responsive patients before and after intervention in the tDCS (sham) + DREC (sham) group.

**Fig 4:**
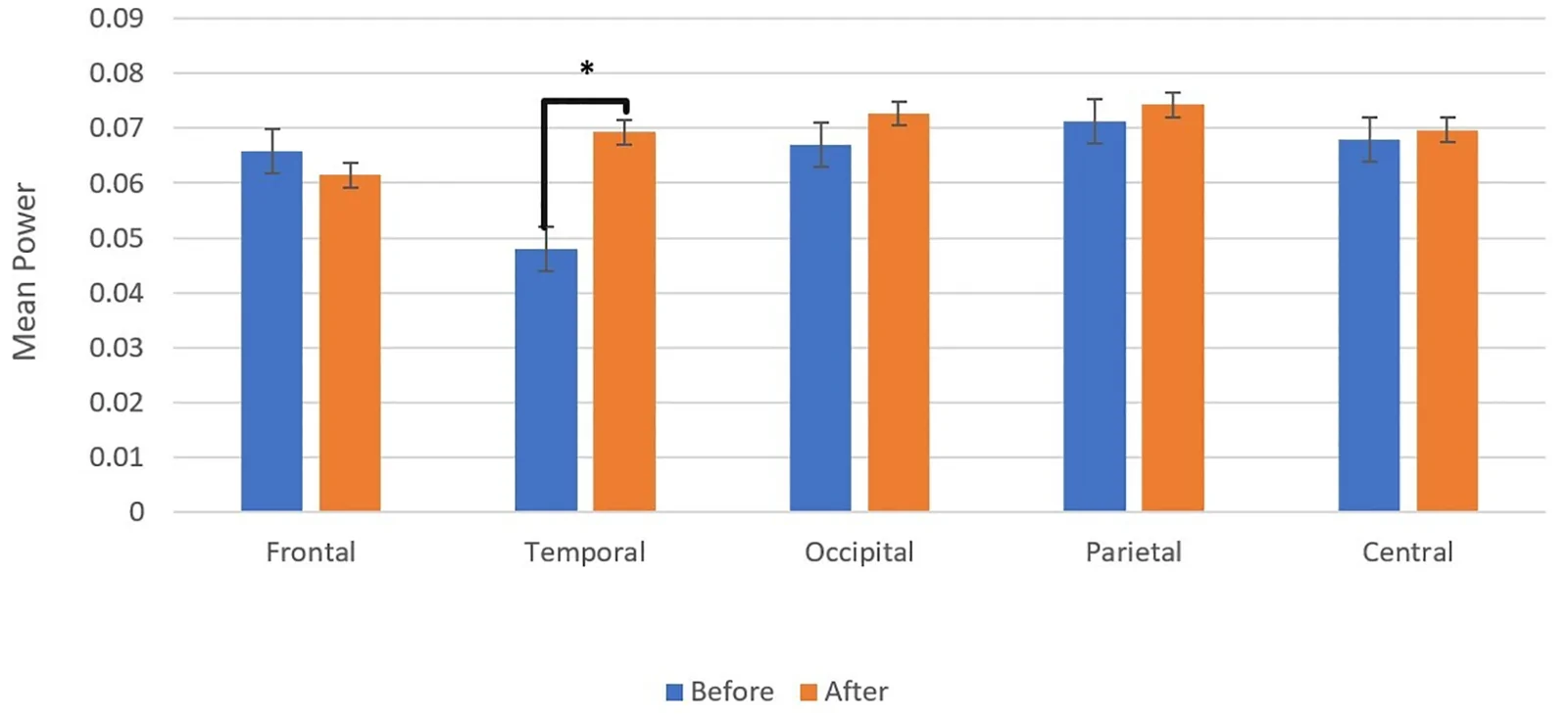
Comparison of mean power in brain lobes in treatment-resistant patients before and after intervention in the tDCS (sham) + DREC (sham) group. * p < 0.05.

It should be noted that in the tDCS (sham) + DREC (sham) intervention group, there were 5 treatment-resistant and 7 treatment-responsive subjects. In the tDCS + DREC (sham) group, only two were treatment-resistant, and the other ten were treatment-responsive. In the other two intervention groups, there were no treatment-resistant subjects and all subjects were improved. Therefore, the treatment-responsive and treatment-resistant individuals in the tDCS (sham) + DREC (sham) group were examined and compared for the average power in each brain region (frontal, parietal, occipital, temporal, and central). The frontal region includes information from channels FP1, FP2, F3, F4, F7, F8 and FZ; occipital includes O1, O2; temporal includes T3, T4, T5 and T6; parietal includes P3, P4 and PZ, and central includes C3, C4 and CZ. Figs 3 and 4 show the difference in mean power in each brain lobe in treatment-responsive and treatment-resistant patients, respectively.

In the treatment-responsive patients in the tDCS (sham) + DREC (sham) group, a non-significant increase in the mean power after the intervention was observed for the central and parietal regions, and a non-significant decrease in the mean power for the occipital and temporal regions, and the frontal lobe power remained almost constant. In the treatment-resistant patients in the tDCS (sham) + DREC (sham) group, a non-significant increase in the mean power after the intervention was observed for the central, parietal, and occipital regions, and a non-significant decrease in the mean power for the frontal region. A significant increase in the mean power after the intervention was observed only in the temporal lobe (p < 0.05).

Notably, the statistical analysis to check whether the changes are significant or not significant, cannot be examined due to the small number of treatment-resistant patients in tDCS + DREC (sham) group. Also, no comparison can be made in other groups due to the lack of treatment-resistant patients.

Previous studies have identified structural and functional differences in the brains of OCD patients who respond differently to treatment, notably in regions such as the orbitofrontal cortex and anterior cingulate cortex [34,35]. On the other hand, one major challenge in applying non-invasive brain stimulation (NIBS) methods to treat psychiatric and neurological disorders is inter- and intra-individual response to NIBS [36]. Therefore, it can be concluded that the patterns created by each intervention differ between treatment-responsive and treatment-resistant patients, and they can be identified and predicted by extracting and selecting appropriate features from the EEG signal and using mathematical modeling.

**Fig 5:**
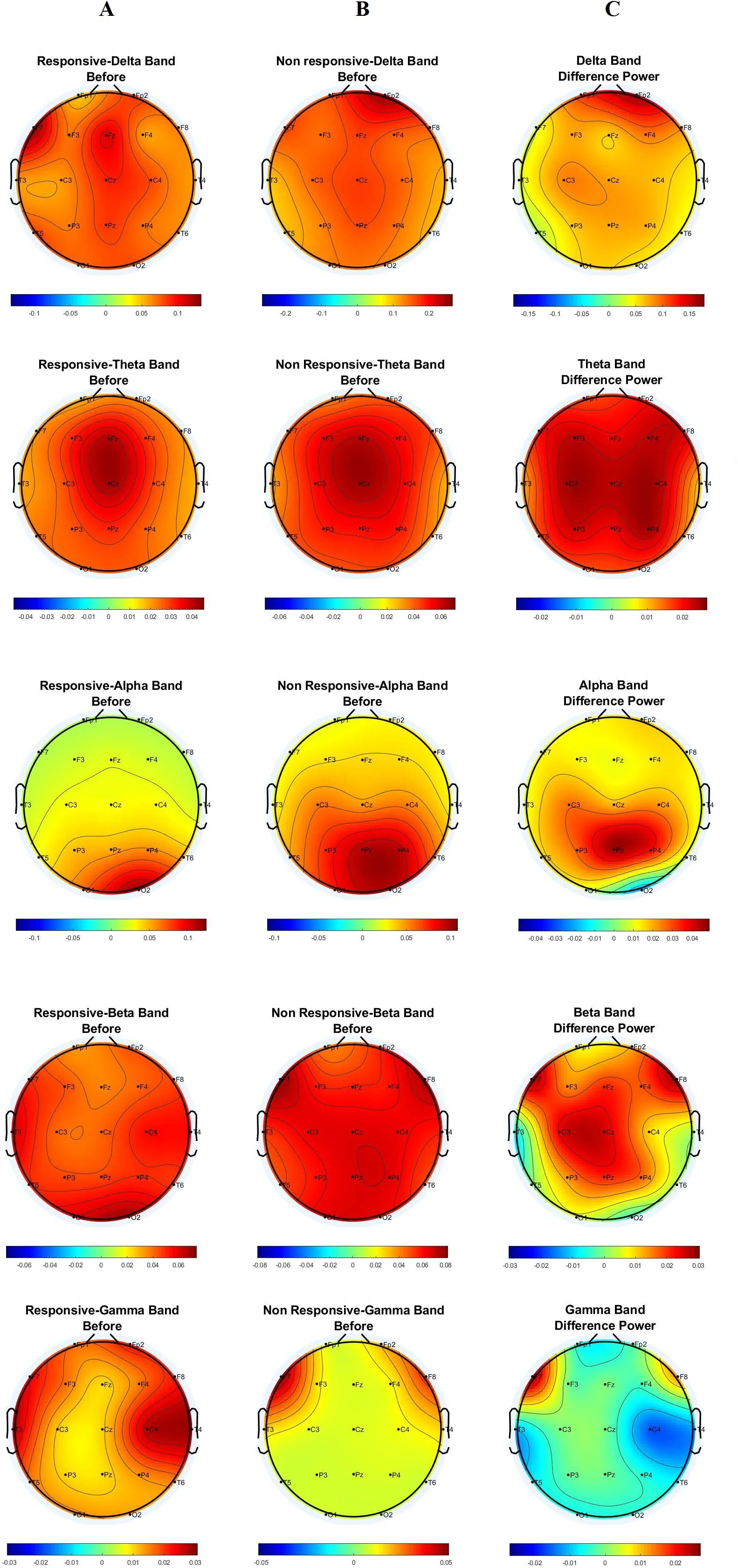
QEEG in the tDCS (sham) + DREC (sham) group for A) Treatment-responsive and B) Treatment-resistant subjects, and C) power difference for each frequency band (delta, theta, alpha, beta and gamma).

Fig 5 shows the QEEG in all frequency spectrum and in the tDCS (sham) + DREC (sham) intervention group. Also, the difference in power between treatment-responsive and treatment-resistant was calculated and plotted in panel C. It is observed a significant increase in signal power before receiving the intervention (tDCS (sham) + DREC (sham)) in treatment-responsive compared to treatment-resistant patients in the gamma spectrum (p < 0.05) and a significant decrease observed in the delta, theta, alpha and beta spectrums (p < 0.05).

On the other hand, the frequent suggestion of the power feature in the ReliefF algorithm, as well as the difference in the baseline power of treatment-responsive and treatment-resistant patients before the intervention, may indicate the importance of this feature in prediction, which is consistent with the results of a previous study [37].

### Model assessment

To select the appropriate features, several linear and nonlinear features were extracted from the EEG signal that comprehensively present the dynamics and patterns of the EEG signal. Then, from among these features, features with higher weights were identified using the ReliefF algorithm. It was observed that the most suggested and repeated features by ReliefF were the median and peak power and FSL, respectively.

In the second step, the appropriate feature was used as input to train the ANN. It should be noted that to improve the accuracy and reduce the error of the proposed models, the models were examined with different parameters and inputs, and the best case for each model in each frequency spectrum and each intervention group was selected by trial and error. For all models, the best performance was in the case where the inputs to the models were the first 3 features with the highest rank proposed by Relief. All models were implemented in MATLAB software. The error calculated was Root Mean Squared Error (RMSE). The fully connected feedforward network achieved the performance with an average RMSE of 2.73 ± 1.06. Also, the RMSE for the RBF model was 2.97 ± 1.44. More details are included in Table 3. Also, K-fold cross-validation was applied to verify the accuracy of the models and to prevent overfitting. Nevertheless, the risk of overfitting cannot be underestimated due to the relatively small sample size.

**Table 3:** Results of fully connected feedforward network, RBF, and GWO+ fully connected feedforward network Models.

Intervention Group	Frequency Band	Fully connected feedforward network RMSE (Mean)	RBF RMSE (Mean)	GWO+ Fully connected feedforward network RMSE(Mean)
**tDCS + DREC (sham)**	Delta	4.50	4.67	1.41
	Theta	3.58	4.25	0.13
	Alpha	4.25	4.50	0.99
	Beta	4.50	6.17	0.99
	Gamma	4.08	5.00	2.08
	**Overall (Mean±SD)**	**4.18 ± 0.38**	**4.91 ± 0.75**	**1.12 ± 0.71**
**DREC + tDCS (sham)**	Delta	2.92	2.92	1.06
	Theta	2.92	2.92	0.52
	Alpha	2.83	2.92	0.31
	Beta	2.25	2.92	0.78
	Gamma	2.42	2.92	0.49
	**Overall (Mean±SD)**	**2.67 ± 0.31**	**2.92**	**0.63 ± 0.29**
**DREC + tDCS**	Delta	2.42	2.83	0.43
	Theta	1.83	1.67	0.07
	Alpha	2.92	2.92	0.63
	Beta	2.58	2.75	0.18
	Gamma	2.58	2.92	0.38
	**Overall (Mean±SD)**	**2.47 ± 0.40**	**2.61 ± 0.53**	**0.34 ± 0.22**
**tDCS (sham)+DREC (sham)**	Delta	1.33	1.42	0.002
	Theta	2.08	1.58	0.03
	Alpha	1.67	1.42	0.12
	Beta	1.58	1.42	0.77
	Gamma	1.42	1.42	0.18
	**Overall (Mean±SD)**	**1.62 ± 0.29**	**1.45 ± 0.07**	**0.22 ± 0.32**
	**Model Average (Mean±SD)**	**2.73 ± 1.06**	**2.97 ± 1.44**	**0.57 ± 0.4**

**Fig 6:**
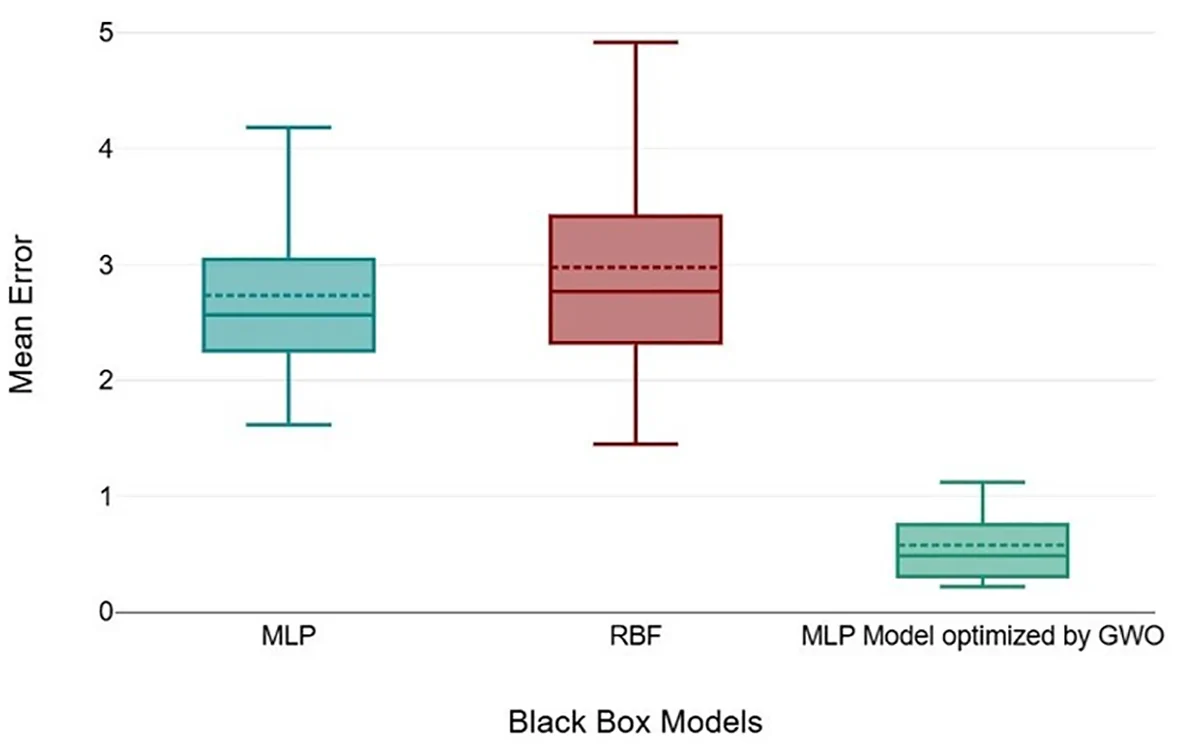
Black-box models’ errors based on the mean of RMSE.

In the next step, to improve the prediction results and reduce the error, the GWO algorithm was used to optimize the fully connected feedforward network. K-fold was also used to verify the accuracy of this algorithm. GWO used to optimized fully connected feedforward network parameters (weight and bias values). The network structure consisted of a hidden layer with 10 neurons, stop criterion was maximum iteration, the number of iterations was 1500, search range was (0,1), and the number of wolves was 50. The fully connected feedforward network optimized by GWO achieved the best predictive performance with an average RMSE 0.57 ± 0.4. Fig 6 shows the black-box models (fully connected feedforward network, RBF and fully connected feedforward network model optimized by GWO) errors based on RMSE. All MATLAB codes used in this study are available in the S1 File.

The results show that the prediction error (RMSE) with the help of the GWO meta-heuristic algorithm has been greatly reduced compared to the other two models (0.57 ± 0.4). Here, similar to the fully connected feedforward network and RBF models, it is observed that the error in the tDCS (sham) + DREC (sham) group is less than the other groups. However, in the tDCS + DREC (sham) group, the error value is maximized. Despite the extraction of nonlinear features from the EEG and the presence of these features in the input of the models, the models have probably not been able to predict the nonlinear changes caused by the tDCS intervention in the brain well.

It should be noted that although there may be differences in the details between individuals, similarities were observed in the pattern of the EEG signal. In addition, a research question was whether these models operate individually or in a generalized manner? In this research, individual model training was achieved by optimizing the model parameters and the results obtained from their output.

## Discussion

A significant challenge in the OCD treatment is the substantial proportion of patients (40–60%) resistant to first-line therapies [11]. The resistance underscores the need for alternative strategies, such as non-invasive brain stimulation [10]. However, the effectiveness of these methods is subject to considerable individual variability [38] and potential interactions with concurrent psychiatric medications [39], which are common due to high comorbidity [40]. It is notable that in this study, the primary objective was to evaluate the proposed concept rather than to address clinical transparency in terms of error. Nevertheless, ΔY-BOCS represents an integer variable, while the minimum mean error optimized by the GWO algorithm was 0.57 ± 0.4. In a potential clinical application, if a clinician seeks to predict a patient’s ΔY-BOCS using EEG signals through an ANN model, the system could provide a reasonably accurate estimation of treatment effectiveness. This would minimize the risk of situations in which a clinician anticipates significant improvement and a substantial reduction in ΔY-BOCS, whereas the patient, after undergoing the intervention and devoting time and resources, experiences deterioration instead. Therefore, predicting individual treatment responses is crucial to alleviate patient burden, costs, and optimize therapeutic selection. This study represents, to our knowledge, the first attempt to predict tDCS effectiveness in C-OCD patients using pre-intervention EEG features and machine learning. For example, a previous study has investigated predictors of pharmacological response using machine learning techniques, relying mainly on sociodemographic, clinical, and cognitive variables such as baseline Y‑BOCS scores, depression severity, and performance on Digit Span and Raven’s Matrices [41]. In contrast, our research emphasizes EEG as an objective neurophysiological signal of treatment response. This key difference highlights the innovation of our method, shifting from self-reports and clinical assessments to brain-based predictors.

The observed placebo response in the sham tDCS and sham DREC group aligns with existing literature [42], highlighting the significant impact of placebo on OCD symptoms and EEG patterns [43,44]. Conversely, the consistent improvement in the active DREC with sham tDCS group suggests that DREC effectively reduces disgust-related symptoms through conditioning.

The variability in feature selection and intervention outcomes reflects the network-based pathology of OCD, where interventions target distinct neural circuits. This heterogeneity emphasizes the importance of personalized predictions over generalized biomarkers. Moreover, the potential for tDCS to modulate brain states and enhance the effects of cognitive interventions, as observed in the combined active tDCS and active DREC group, warrants further investigation [45].

Brain stimulation may cause changes in the brain state by changing the synchronization patterns in the brain and make it ready for change. In this case, it is likely that if other cognitive interventions (such as DREC in this study) are applied alongside brain stimulation, they will create changes in the brain more easily, and the changes may be more durable, which requires follow-up. For this reason, a combined intervention based on brain stimulation has much greater potential for the effectiveness of the second intervention than an intervention that does not involve brain stimulation, because on the synchronized patterns of the brain and the change in the brain state. In this study, it was also observed that all individuals in the DREC + tDCS group were treatment-responsive, and the reason for this is the change in the brain state by electrical stimulation.

The interventions used in this study each affect specific brain processes and, consequently, specific characteristics of the EEG signal, and therefore, predicting the effectiveness or possibility of effectiveness for each intervention requires its own processing strategies, characteristics, and model. In addition, all of these can be completely dependent on the state of each person’s brain before starting treatment.

As mentioned, among the three proposed models, the mean error of fully connected feedforward network optimized by GWO, was reported to be lower than the other models (0.57 ± 0.4). In all three black-box models, the error rate in the tDCS (sham) + DREC (sham) group was lower than the other intervention groups. Also, the highest error rate reported was related to the tDCS + DREC (sham) intervention group.

One reason for the lower error in the tDCS (sham) + DREC (sham) group could be the higher number of treatment-resistant individuals compared to the other groups, potentially due to a more balanced training dataset. Because in the other groups, treatment-resistant individuals either do not exist or there are only two individuals, which is a small number for training.

Regarding the maximum error in all models was in the tDCS + DREC (sham) group, it seems that despite extracting nonlinear features from the EEG and the presence of these features in the model input, the models may not have been able to predict the nonlinear changes caused by the tDCS intervention in the brain well. Of course, it is not far-fetched that the functional connectivity index of the brain in specific networks in the brain is affected by tDCS. Previous studies have also shown the effect of tDCS on the functional connections of the brains of people with OCD and the improvement of their symptoms [46].

ReliefF feature selection consistently identified median and peak power, as well as FSL (related to functional connectivity), as key predictors. The reason for the FSL suggestion by ReliefF could be that the connections and consequently the brain synchrony feature, especially in the tDCS group, were more affected by this intervention, and hence it was suggested by ReliefF. The suggestion of the EEG power as a distinguishing feature by ReliefF algorithm may indicate the importance of power in prediction, this aligns with previous researches highlighting the importance of EEG power in predicting treatment response [37,47–49]. For example in [37], the prediction of the effectiveness of TMS intervention in OCD patients was investigated using features extracted from QEEG power spectrum features and machine learning methods (ANN and Particle Swarm Optimization (PSO) algorithm). The definition of treatment-responsive in this study was based on the psychiatrist’s evaluation, and the Y-BOCS questionnaire was not used. This study showed that among the 4 EEG bands (delta, theta, alpha and beta), theta power (4–8 Hz) with 80% accuracy was more able to distinguish treatment-responsive patients from treatment-resistant patients than other frequency bands, and treatment-responsive OCD patients had higher theta power in all electrodes than treatment-resistant patients [37]. In other words, the purpose of the study was to obtain biomarkers to predict and classify treatment-responsive and treatment-resistant patients. In this way, prediction requires comparing treatment-responsive and treatment-resistant patients’ power data.

As mentioned in the description of Fig 5, the treatment-responsive patients in tDCS (sham) + DREC (sham) group had a significantly higher gamma and a significantly lower delta, theta, alpha, and beta power before the intervention compared to treatment-resistant. In a previous study, prediction of the effect of paroxetine in OCD patients done by QEEG and a significant increase in gamma power was observed before the start of treatment in early treatment-resistant individuals [50]. So, our study shows different and controversial results in comparison to the mentioned research.

Notably, QEEG can only present the prediction in the form of a classification. In other words, the treatment-responsive and resistant patients’ data in each intervention group must be present to compare, otherwise, the prediction cannot be made. So, the disadvantage of this method is that the prediction for one person cannot be made independently of other individuals. Additionally, the prediction of the level of improvement is uncertain. Under these circumstances, the importance of the individualized models, that can predict independently of other individuals in the intervention group and before the start of treatment, becomes more apparent.

By comparing EEG signals before and after the intervention, features were identified that showed marked changes following the intervention. Changes in linear and nonlinear EEG features can reflect changes in interactions between brain regions and synchronization patterns, representing the brain’s electrophysiological response to the interventions. The relationship between selected EEG features and changes in Y-BOCS scores was identified by the ANN, enabling accurate prediction of intervention outcomes. These possible mechanisms are proposed as preliminary explanations and should be further examined in future studies. It should be noted that previous literature indicates that various interventions may influence multiple neurophysiological mechanisms, including GABAergic, glutamatergic, and noradrenergic systems. Investigating such precise mechanisms requires experimental animal studies.

It should be noted that one of the main limitations of this study is the small sample size in each group. Also, another limitation is the absence of two other groups in this study (6 groups instead of 4 groups), in which one group received only the tDCS intervention and the other group received only the DREC intervention, so that the effect of each intervention could be measured alone and without the interference of the placebo or nocebo effect of the other intervention.

## Conclusion

This study demonstrates the feasibility of predicting tDCS efficacy in C-OCD using pre-intervention EEG features and machine learning. The fully connected feedforward network-GWO model exhibited promising performance, suggesting its potential for individualized treatment planning. So, it can prevent the high costs of ineffective treatments in C-OCD patients. Nevertheless, relatively small group sizes and imbalanced responder distributions across intervention groups might affect the performance of machine learning algorithms such as ANN. It has to be addressed in the next studies. Thus, future studies with larger cohorts are needed to validate and refine these findings.

## Supporting information

S1 Appendix Equations used in the study. (DOCX)

S1 File MATLAB codes. (ZIP)

S2 File Pre- and post-intervention Y-BOCS scores (used for statistical analysis). (PDF)

S3 File CONSORT checklist. (PDF)

S4 File Trial study protocol. (PDF)

S1 Dataset Anonymized EEG data (Part1). (RAR)

S2 Dataset Anonymized EEG data (Part2). (RAR)
