# Supplementary material for: Evaluating the performance of EEG based on ANN to predict the effectiveness of tDCS combined evaluative conditioning on obsession symptoms reduction in contamination OCD patients: Secondary analysis of data from a randomized controlled trial
Source: PLoS One. 2026 Aug 20;21(8):e0354614. doi: 10.1371/journal.pone.0354614 (PMC13492803; doi:10.1371/journal.pone.0354614)
Supplement: Supplementary file 1 [file pone.0354614.s003.pdf]

S4 File. Pre- and post-intervention Y-BOCS scores (used for statistical analysis).

ID	Group	Pre_YBOCS	Post_YBOCS	YBOCS_Change
1	1	17.00	17.00	0.00
2	1	25.00	24.00	-1.00
3	1	21.00	19.00	-2.00
4	1	17.00	15.00	-2.00
5	1	23.00	24.00	1.00
6	1	31.00	29.00	-2.00
7	1	25.00	24.00	-1.00
8	1	25.00	26.00	1.00
9	1	22.00	21.00	-1.00
10	1	27.00	27.00	0.00
11	1	22.00	20.00	-2.00
12	1	33.00	39.00	6.00
13	2	31.00	26.00	-5.00
14	2	28.00	14.00	-14.00
15	2	20.00	8.00	-12.00
16	2	20.00	11.00	-9.00
17	2	30.00	26.00	-4.00
19	2	26.00	31.00	5.00
19	2	24.00	25.00	1.00
20	2	28.00	26.00	-2.00
21	2	24.00	23.00	-1.00
22	2	30.00	28.00	-2.00
23	2	27.00	18.00	-9.00
24	2	30.00	27.00	-3.00
25	3	26.00	19.00	-7.00
26	3	22.00	15.00	-7.00
27	3	26.00	22.00	-4.00
28	3	23.00	16.00	-7.00
29	3	21.00	19.00	-2.00
30	3	21.00	14.00	-7.00
31	3	21.00	10.00	-11.00
32	3	27.00	15.00	-12.00
33	3	21.00	20.00	-1.00
34	3	31.00	24.00	-7.00
35	3	21.00	14.00	-7.00
36	3	25.00	15.00	-10.00
37	4	27.00	19.00	-8.00
38	4	29.00	20.00	-9.00

39	4	30.00	18.00	-12.00
40	4	25.00	22.00	-3.00
41	4	19.00	16.00	-3.00
42	4	31.00	24.00	-7.00
43	4	25.00	22.00	-3.00
44	4	23.00	19.00	-4.00
45	4	26.00	24.00	-2.00
46	4	19.00	16.00	-3.00
47	4	28.00	21.00	-7.00
48	4	21.00	15.00	-6.00
