# Supplementary material for: Evaluating the performance of EEG based on ANN to predict the effectiveness of tDCS combined evaluative conditioning on obsession symptoms reduction in contamination OCD patients: Secondary analysis of data from a randomized controlled trial
Source: PLoS One. 2026 Aug 20;21(8):e0354614. doi: 10.1371/journal.pone.0354614 (PMC13492803; doi:10.1371/journal.pone.0354614)
Supplement: Supplementary file 3 [file pone.0354614.s005.pdf]

This study is a four-arm, randomized, placebo-controlled clinical trial in which participants get sham or real forms of two interventions, including EC and tDCS. It is aimed to investigate the effectiveness of EC, tDCS, and a combination of both on disgust reduction, clinical symptoms, and cognitive characteristics in C-OCD patients. Randomly assignment will be in blocks of 4 in 1:1:1:1 allocation to four parallel groups: (1) real EC plus real tDCS; (2) sham EC plus real tDCS; (3) real EC plus sham tDCS; (4) sham EC plus sham tDCS. We consider sham interventions to be able to separate the exposure effect from the EC effect and also to monitor the placebo effect. Assessments occur at baseline, after completion of 10 interventional sessions, and in a 2-month follow-up. All groups have similar assessments and equal intervention amounts (equal time, activity, and number of sessions).

Figure 1 shows the design of the study. The trial will be conducted in the Cognitive Science Laboratory of Ferdowsi University of Mashhad, Mashhad, Iran.

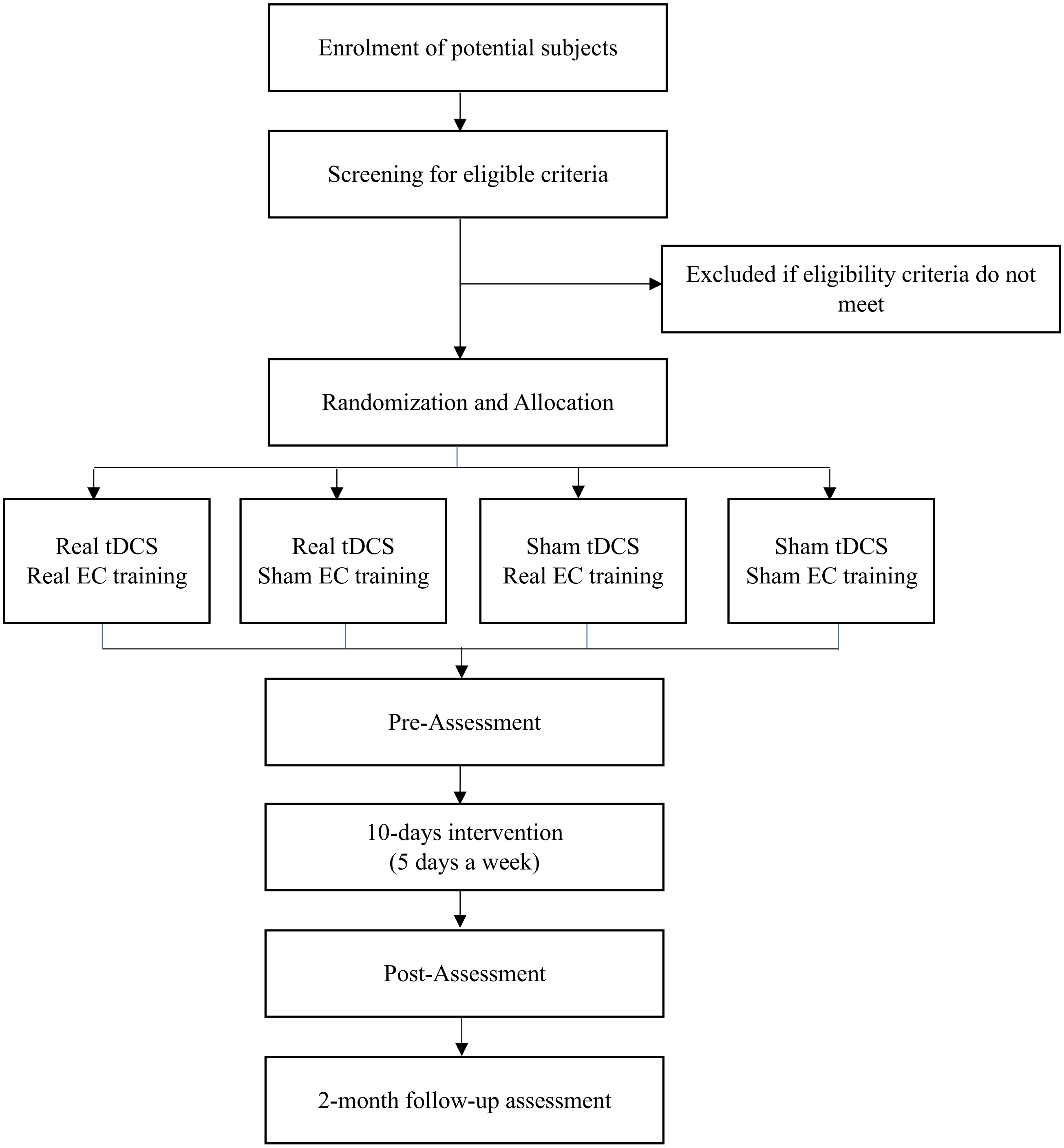

Fig. 1 CONSORT flow chart.
