# Supplementary material for: Evaluating the performance of EEG based on ANN to predict the effectiveness of tDCS combined evaluative conditioning on obsession symptoms reduction in contamination OCD patients: Secondary analysis of data from a randomized controlled trial
Source: PLoS One. 2026 Aug 20;21(8):e0354614. doi: 10.1371/journal.pone.0354614 (PMC13492803; doi:10.1371/journal.pone.0354614)
Supplement: Supplementary file 4 [file pone.0354614.s001.docx]

**S1 Appendix:** **Equations**

A) Power spectrum:

The discrete Fourier transform for a discrete-time signal x[n] is as follows:

(1)

$$X\left( m \right)=\sum_{n=0}^{N-1} x\left[ n \right] exp\left( \frac{-j2\pi mn}{N} \right) m=0,1,2,3,\ldots\ldots\ldots\ldots\ldots\ldots\ldots N-1$$

Where j is the imaginary unit (j² = -1), m is the frequency bin index, n is the time sample index, and N is the total number of samples.

To obtain the power spectrum of this signal, the following equation is used:

(2)

$\hat{P}_{per}\left( f_{m} \right)=\frac{1}{N\Delta t}\left| X\left[ m \right] \right|^{2} \Delta t=\frac{1}{f_{s}}$

Where *N* is the number of samples in the signal, ∆t is the sampling period, and *f_s_* is the sampling frequency (1).

B) FSL:

Trajectory reconstructions based on chaos theory require the determination of the time delay (τ) and the dimension of the Embedding dimension (*d*) to form the state space. In such a state space, the n^th^ state *x_k,n_* of the k^th^ trajectory *x_k_* is given by:

(3)

$$\boldsymbol{X}_{\boldsymbol{k,n}}\boldsymbol{=}\left( \boldsymbol{x}_{\boldsymbol{k,n}}\boldsymbol{,}\boldsymbol{x}_{\boldsymbol{k,n+}\boldsymbol{\tau}}\boldsymbol{,\ldots,}\boldsymbol{x}_{\boldsymbol{k,n+}\left( \boldsymbol{d-}\boldsymbol{1} \right)\boldsymbol{\tau}} \right)$$

where *x_k,n_* is the n^th^ point of the kth time series (*k=1,2,…,M*; which is considered as the number *M* of the time series *x_k,n_*. Here *k* is the number of EEG channels and n is the number of samples)(2).

The probability that two vectors *x_k,m_* and *x_k,n_* are closer than a distance ε to each other in the embedding space, if *w1 <|n–m|<w2*, is obtained from the following relationship:

(4)

$$\boldsymbol{P}_{\boldsymbol{k,n}}^{\boldsymbol{ℇ}_{\boldsymbol{k,n}}}\boldsymbol{=}\frac{\boldsymbol{1}}{\boldsymbol{2(w2-w1)}}\sum_{\begin{aligned} \boldsymbol{m=1} \\ \boldsymbol{w1<|n-m|<w2} \end{aligned}}^{\boldsymbol{N}} \boldsymbol{\Theta}\left( \boldsymbol{ℇ}_{\boldsymbol{k,n}}\boldsymbol{-}\left| \boldsymbol{Xk,n-Xk,m} \right| \right)$$

In this relation, Θ is the Heaviside step function and | | represents the Euclidean distance and *N* the number of states of each trajectory. *w1* is the Theiler correction to avoid Information redundancy in similar calculations. *w2*, expressed as an integer, is the maximum time distance that a state vector can have from the reference state. In fact, considering that here each time series is considered as a time window, *w1* and *w2* are the beginning and end of the time window, respectively. P_ref_= 0.05 is considered for all-time series. Also, *μ_k,n_* (*X_k,m_*) is the membership value for state *X_k,m_* in the Gaussian function with dimensions d (the dimension of the reconstruction space) of the k^th^ time series.

By establishing the condition P_ref_ << S_k,n_<<1, a critical distance *ε_k,n_* is obtained for *X_k,n_* as a neighborhood boundary, and then the number of time series whose states *X_k,m_* and *X_k,n_* are closer than *ε_k,n_* in a Euclidean sense will be calculated from the following equation:

(5)

$$\boldsymbol{H}_{\boldsymbol{n,m}}\boldsymbol{=}\sum_{\boldsymbol{k=}\boldsymbol{1}}^{\boldsymbol{M}} \boldsymbol{\Theta}\left( \boldsymbol{ℇ}_{\boldsymbol{k,n}}\boldsymbol{-}\left| \boldsymbol{X}\boldsymbol{k,n-}\boldsymbol{X}\boldsymbol{k,m} \right| \right)^{\boldsymbol{1}}$$

Then the similarity of the k^th^ time series (*k=1,2,…,M*) to *M-1* other time series, and for each pair (*X_k,m_* and *X_k,n_*) is calculated as follows:

(6)

$$\boldsymbol{S}_{\boldsymbol{k,n,m}}\boldsymbol{=}\frac{\boldsymbol{H}_{\boldsymbol{n}\boldsymbol{,}\boldsymbol{m}}\boldsymbol{-1}}{\boldsymbol{M-1}}\boldsymbol{\Theta}\left( \boldsymbol{ℇ}_{\boldsymbol{k,n}}\boldsymbol{-}\left| \boldsymbol{X}\boldsymbol{k,n-}\boldsymbol{X}\boldsymbol{k,m} \right| \right)$$

Then, by averaging over discrete time samples, the SL measure will be as follows:

(7)

$$S_{k,n}=\frac{\sum_{\begin{aligned} m=1 \\ w1<|n-m|<w2 \end{aligned}}^{N} S_{k,n,m}}{\sum_{\begin{aligned} m=1 \\ w1<|n-m|<w2 \end{aligned}}^{N} \Theta\left( ℇ_{k,n}-\left| Xk,n-Xk,m \right| \right)}$$

The next steps will be to generalize this relationship using fuzzy logic rules.

First, a Gaussian membership function centered at the reference state and with standard deviation *ε_k,n_* is considered as equation 8:

(8)

$$\mu_{k,n} (X_{k,m}) =exp(-\frac{\left| Xk,n-Xk,m \right|^{2}}{ℇ_{k,n}})$$

In the above equation, *μ_k,n_* (*X_k,m_*) is the membership value for state (*X_k,m_*)in the Gaussian function with dimensions *d* of the k^th^ time series.

Substituting the Heaviside step function into equation 4, we have:

(9)

$$P_{k,n}^{ℇ_{k,n}}=\frac{1}{2(w2-w1)}\sum_{\begin{aligned} m=1 \\ w1<|n-m|<w2 \end{aligned}}^{N} \mu_{k,n}\left( X_{k,m} \right)$$

For all values of k, by considering$P_{k,n}^{ℇ_{k,n}}$ equal to a small P_ref_ <<1, $ℇ_{k,n}$is obtained. Here, considering the Gaussian membership function, the similarity value of each state to the reference state changes between zero and one, and the value of the distances will vary between zero and infinity. The number of time series of states *X_k,m_* and *X_k,n_* in the embedding space in the FSL method will be as follows:

(10)

$$H_{n,m}=\sum_{k=1}^{N} \mu_{k,n}\left( X_{k,.m} \right)$$

In this regard, unlike equation 5, where the Heaviside step function was used and produced definite results of 0 and 1 in the output, in the above equation, the presence of Gaussian membership has created such a problem. Considering this point and equation 7 and equation 8, the probability index will be as follows:

(11)

$$S_{k,n}=\frac{\sum_{\begin{aligned} m=1 \\ w1<|n-m|<w2 \end{aligned}}^{N} [(\frac{{H_{n,m}-\mu}_{k,n}(X_{k,m})}{M-1}){.\mu}_{k.n}(X_{k,m})]}{\sum_{\begin{aligned} m=1 \\ w1<|n-m|<w2 \end{aligned}}^{N} \mu_{k,n}\left( X_{k,m} \right)^{1}}$$

Here, as in the last part of calculating the final SL relationship, averaging is used and the final FSL relationship is obtained as the following relationship (2):

(12)

$$S_{2,n}=\frac{1}{2 P_{ref}(w2-w1)}\sum_{\begin{aligned} m=1 \\ w1<|n-m|<w2 \end{aligned}}^{N} [{\mu_{1,n}(X_{1,m})\mu}_{2,n}(X_{2,m})]$$

**References:**

1.	Akin M, Kiymik MK. Application of Periodogram and AR Spectral Analysis to EEG Signals. Journal of Medical Systems. 2000 Aug 1;24(4):247–56.

2.	Ahmadlou M, Adeli H. Fuzzy synchronization likelihood with application to attention-deficit/hyperactivity disorder. Clin EEG Neurosci. 2011 Jan;42(1):6–13.
